# A new *Otacilia* Thorell, 1897 species from Hubei Province, China (Araneae, Phrurolithidae)

**DOI:** 10.3897/BDJ.12.e137014

**Published:** 2024-10-23

**Authors:** Minghao Guo, Yannan Mu, Feng Zhang

**Affiliations:** 1 Hebei Vocational University of Industry and Technology, Shijiazhuang, China Hebei Vocational University of Industry and Technology Shijiazhuang China; 2 Key Laboratory of Zoological Systematics and Application, College of Life Sciences, Hebei University, Baoding, China Key Laboratory of Zoological Systematics and Application, College of Life Sciences, Hebei University Baoding China; 3 Hebei Basic Science Center for Biotic Interaction, Hebei University, Baoding, China Hebei Basic Science Center for Biotic Interaction, Hebei University Baoding China

## Abstract

**Background:**

Phrurolithidae is a family of spiders with 405 species belonging to 25 genera distributed worldwide. Notably, 213 species belonging to 17 genera have been recorded in China.

**New information:**

A new species of the genus *Otacilia* Thorell, 1897 is described from Duheyuan Nature Reserve, Hubei Province, China. Diagnosis, morphological description, living photos and photos of the habitus and genitalia of the new species are provided.

## Introduction

*Otacilia* Thorell, 1897, the largest genus of family Phrurolithidae, contains 143 species and is distributed in East Asia and Southeast Asia; amongst them, 120 species were reported in China (Anonymous 2024). The species and studies of *Otacilia* have accelerated considerably during past decade and reduced the complexity of *Otacilia* by assigning species to newly-established genera (Liu et al. 2020, Zamani and Marusik 2020, Kamura 2021, Mu and Zhang 2021, Liu et al. 2022, Mu and Zhang 2022, Mu et al. 2022, Mu and Zhang 2023), which greatly promoted the study of *Otacilia*. Recently, *O.khezu* Lin & Li, 2024, a eyeless species collected from a cave was described, showing enormous potential for species diversity (Lin et al. 2024). While examining specimens collected from Duheyuan Nature Reserve, one new *Otacilia* species has been discovered and is described here: *Otaciliasubshanxi* **sp. nov.**

## Materials and methods

All measurements in the text are given in millimetres. The leg measurements are shown as total length (femur, patella, tibia, metatarsus, tarsus). The epigynes were removed and cleared in a pancreatin solution (Álvarez-Padilla and Hormiga 2007) and then transferred to 95% ethanol. All specimens are preserved in 95% alcohol. Photographs were taken using the Leica M205A stereomicroscope, equipped with a DFC 550 CCD. All specimens are deposited in the Museum of Hebei University (MHBU), Baoding, China.

The abbreviations of genital structures are listed under figure legends. The following abbreviations are used in text: AER—anterior eye row; ALE—anterior lateral eye; AME—anterior median eye; CH—clypeal height; CRW—cephalic region width; CW—carapace width; EAW—eye area width; MOA—median ocular area; PLE—posterior lateral eye; PME—posterior median eye; PER—posterior eye row. Spination: d—dorsal; pl—prolateral; pv—prolateral ventral; rv—retrolateral ventral.

## Taxon treatments

### Otacilia subshanxi sp. nov.

363150EC-BF5E-57EA-9696-EEA5BCDABF0D

D1195279-905F-403F-BDED-986692B2B4C0

#### Materials

- **Type status:** Holotype. **Occurrence:** sex: male; lifeStage: adult; occurrenceID: E556732B-3666-537F-AB57-06D34F340423; **Taxon:** scientificName: *Otaciliasubshanxi*; order: Araneae; family: Phrurolithidae; genus: Otacili; **Location:** country: China; stateProvince: Hubei; county: Zhushan; locality: Shunshuiping Villag; verbatimLatitude: 31°33'7.1359″N; verbatimLongitude: 110°1'10.4883″E; **Event:** year: 2023; month: 9; day: 19
- **Type status:** Paratype. **Occurrence:** sex: 1 male, 5 females; lifeStage: adult; occurrenceID: 74251D57-B22A-59A7-B2A8-7AFE3F008FFE; **Taxon:** scientificName: *Otaciliasubshanxi*; order: Araneae; family: Phrurolithidae; genus: Otacili; **Location:** country: China; stateProvince: Hubei; county: Zhushan; locality: Shunshuiping Village; verbatimLatitude: 31°33'7.1359″N; verbatimLongitude: 110°1'10.4883″E; **Event:** year: 2023; month: 9; day: 19

#### Description

Male (Holotype): total length 3.04, carapace 1.50 long, 1.30 wide; abdomen 1.54 long, 1.03 wide. Eye sizes and interdistances: AME 0.08, ALE 0.09, PME 0.08, PLE 0.10; AME–AME 0.05, AME–ALE 0.01, ALE–ALE 0.22, PME–PME 0.10, PME–PLE 0.06, PLE–PLE 0.40, ALE–PLE 0.08. EAW 0.52, CRW 0.68, EAW/CRW 0.76, CRW/CW 0.52. MOA 0.26 long, anterior width 0.21, posterior width 0.27. CH 0.11, CH/AME 1.38. Labium 0.15 long, 0.22 wide. Sternum 0.87 long, 0.78 wide. Leg measurements: Ⅰ 6.15 (1.57 + 0.55 + 1.81 + 1.48 + 0.74), Ⅱ 4.91 (1.30 + 0.51 + 1.27 + 1.15 + 0.68), Ⅲ 4.02 (1.08 + 0.45 + 0.80 + 1.07 + 0.62), Ⅳ 6.51 (1.78 + 0.54 + 1.53 + 1.80 + 0.86). Spination: femur I d 1 pl 4, femur Ⅱ d 1 pl 2, femur Ⅲ–Ⅳ d 1, tibia Ⅰ pv 7 rv 8, tibia Ⅱ pv 7 rv 6, metatarsus Ⅰpv 4 rv 4, metatarsus Ⅱ pv 4 rv 3.

**Figure 1.**
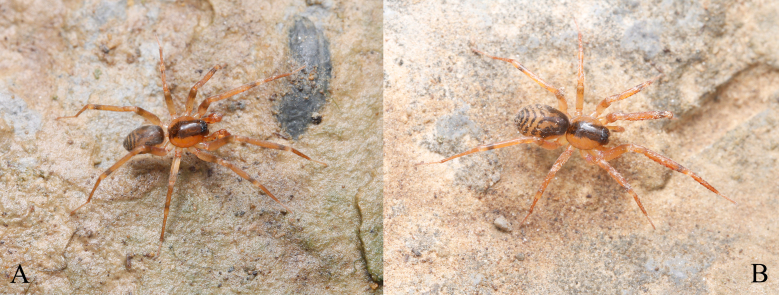
Living photos of *Otaciliasubshanxi* sp. nov.: **A** Male; **B** Female (photographs by Qianle Lu).

**Figure 2.**
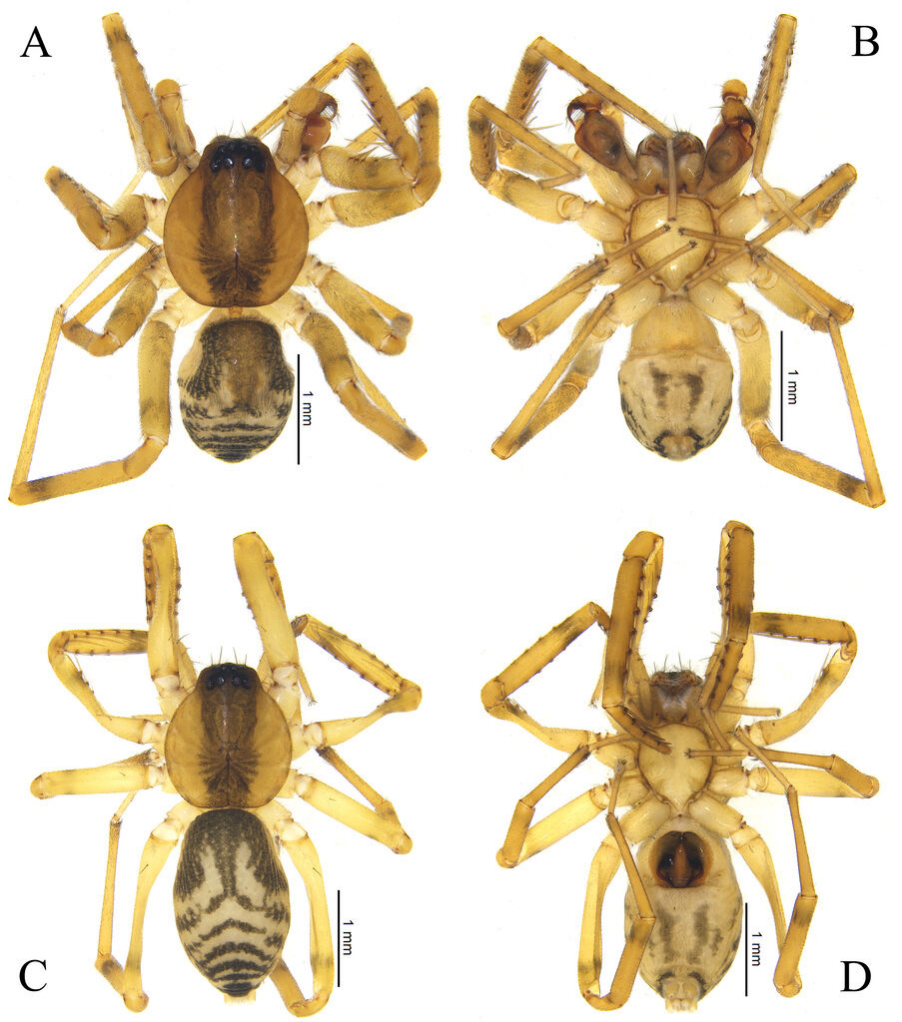
Habitus of *Otaciliasubshanxi* sp. nov.: **A** Male holotype, dorsal view; **B** Same, ventral view; **C** Female paratype, dorsal view; **D** Same, ventral view.

Colouration (Fig. 1A, Fig. 2A and B). Carapace slightly brown, radial striae indistinct, with one black longitudinal stripe nearly same width as eye area. Abdomen grey, with small dorsal scutum darker than carapace, with black pattern beside dorsal scutum anteriorly and four black transverse stripes at posterior of abdomen. Legs yellow, with black annuli near ventral of tibiae I–Ⅳ tip.

**Figure 3.**
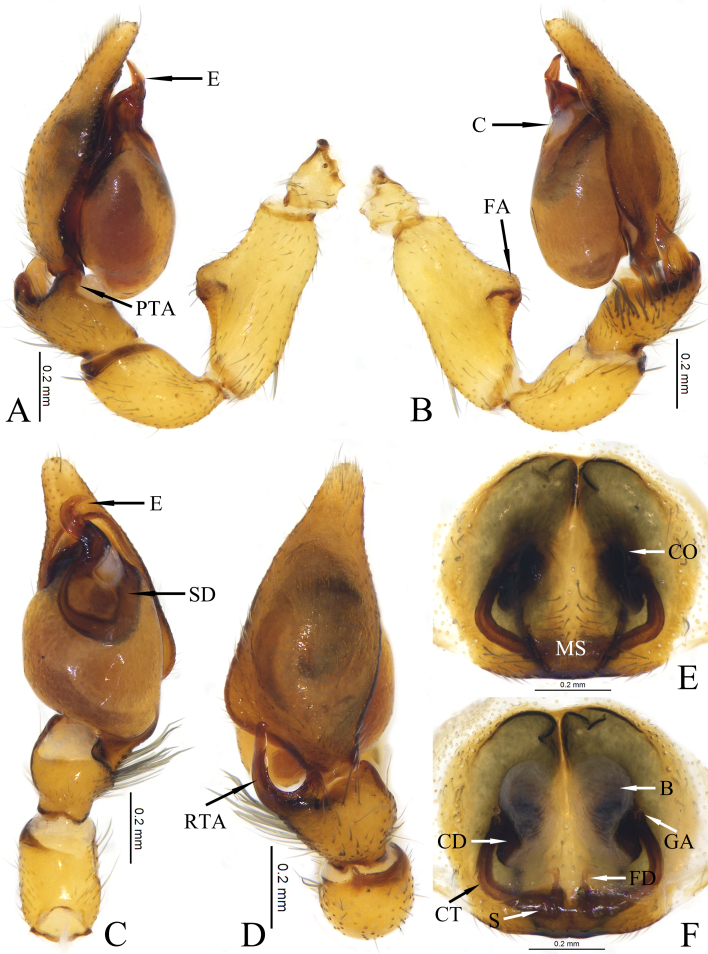
Copulatory organs of *Otaciliasubshanxi* sp. nov.: **A** Male left palp, prolateral view; **B** Same, retrolateral view; **C** Same, ventral view; **D** Same, dorsal view; **E** Epigyne, ventral view; **F** Same, dorsal view. Abbreviations: E—embolus; FA—femoral apophysis; PTA—prolateral tibial apophysis; RTA—retrolateral tibial apophysis; SD—sperm duct; B—bursa; CO—copulatory opening; CD—copulatory duct; CT—connecting tube; FD—fertilisation duct; GA—glandular appendage; MS—median septum; S—spermathecae.

Palp as in Fig. 3A–D. Femur with large, well-developed apophysis at middle part. Prolateral tibial apophysis distinct. Tibial nearly as long as wide. Retrolateral tibial apophysis (RTA) with wide base and narrow, tip blunt, base of retrolateral with a small tuber (Fig. 3D), a row of strong setae at base of RTA (Fig. 3B). Bulb pyriform, sperm duct distinct, tapering off close to embolus. Embolus wide, hook-like, blade-shaped. Conductor small, triangular, membranous.

Female (Paratype): total length 3.52, carapace 1.52 long, 1.36 wide; abdomen 2.00 long, 1.22 wide. Eye sizes and interdistances: AME 0.09, ALE 0.10, PME 0.08, PLE 0.09; AME–AME 0.04, AME–ALE 0.01, ALE–ALE 0.21, PME–PME 0.10, PME–PLE 0.06, PLE–PLE 0.37, ALE–PLE 0. 06. EAW 0.48, CRW 0.69, EAW/CRW 0.69, CRW/CW 0.51. MOA 0.27 long, anterior width 0.19, posterior width 0.27. CH 0.09, CH/AME 1.00. Labium 0.15 long, 0.25 wide. Sternum 0.93 long, 0.79 wide. Leg measurements: Ⅰ 6.00 (1.50 + 0.52 + 1.85 + 1.44 + 0.69), Ⅱ 4.93 (1.25 + 0.51 + 1.33 + 1.13 + 0.71), Ⅲ 4.29 (1.14 + 0.47 + 0.93 + 1.12 + 0.63), Ⅳ 6.32 (1.68 + 0.59 + 1.37 + 1.77 + 0.91). Spination:femur I d 1 pl 4, femur Ⅱ d 1 pl 3, femora Ⅲ–Ⅳ d 1, tibia Ⅰ pv 7 rv 8, tibia Ⅱ pv 7 rv 6, metatarsus Ⅰ pv 4 rv 4, metatarsus Ⅱ pv 4 rv 3. Other characters as in male, except dorsal scutum absent (Fig. 1B, Fig. 2C and D).

Epigyne as in Fig. 3E and F. Epigynal plate sclerotised, non-transparent, with two large atriums. Median septum wide, edge arched, widest at middle part. Copulatory openings located at middle part of atrium, separated by septum. Copulatory ducts short and thick, straight. Connecting tubes long and thin, curved. Bursae balloon-shaped, transparent. Spermathecae oval and small, bean-shaped. Fertilisation ducts short, located anteromesally on spermathecae.

#### Diagnosis

This new species resembles *O.shanxi* Mu & Zhang, 2021 in having a similar femoral apophysis, atrium, curved connecting tubes, but it can be recognised by: 1) the wide embolus (vs. thin, cf. Fig. 3C and fig. 7F in Mu and Zhang 2021), 2) the thin retrolateral tibial apophysis (vs. wide, cf. Fig. 3B and fig. 7G in Mu and Zhang (2021)) and 3) the thin median septum (vs. wide, cf. Fig. 3E and fig. 7F in Mu and Zhang (2021)).

#### Etymology

This species is named for its similarity to *O.shanxi* Mu & Zhang, 2021.

#### Distribution

**Figure 4.**
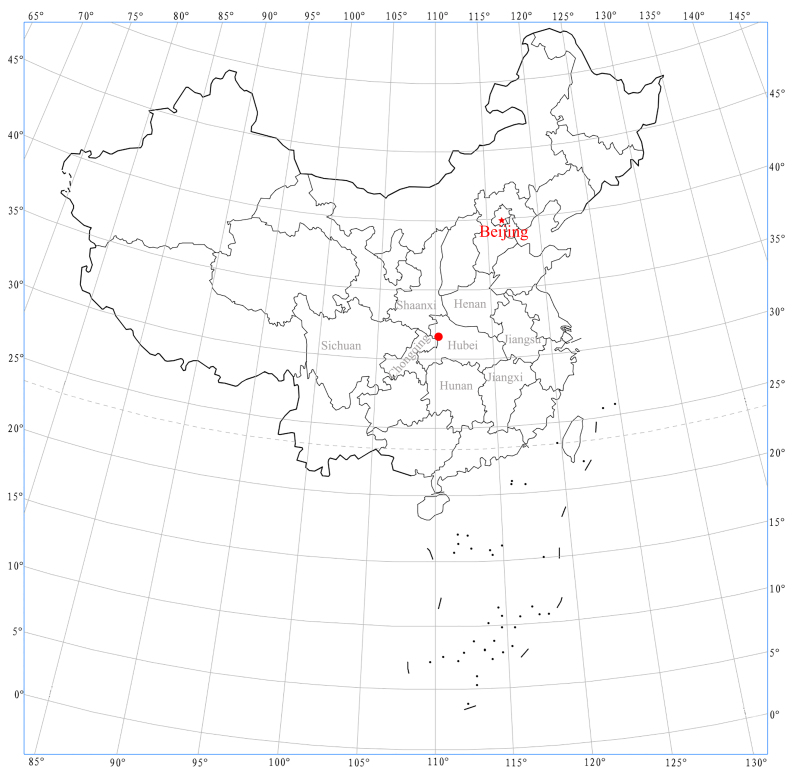
Distribution map of *Otaciliasubshanxi* sp. nov. in this study (red circle).

Know only from the type locality (Fig. 4).

## Supplementary Material

XML Treatment for Otacilia subshanxi
